# Editorial: Intrauterine growth restriction: screening and outcomes

**DOI:** 10.3389/fphys.2024.1438381

**Published:** 2024-06-07

**Authors:** Claire Stenhouse, F. Xavier Donadeu, Fernanda R. C. L. Almeida

**Affiliations:** ^1^ Department of Animal Science, Pennsylvania State University, University Park, PA, United States; ^2^ Division of Translational Bioscience, The Roslin Institute and Royal (Dick) School of Veterinary Studies, University of Edinburgh, Midlothian, United Kingdom; ^3^ Laboratory of Structural Biology and Reproduction, Institute of Biological Sciences, Federal University of Minas Gerais, Belo Horizonte, MG, Brazil

**Keywords:** intrauterine growth restriction, pregnancy, physiology, fetal development, placenta

Intrauterine growth restriction (IUGR) is a condition in which the fetus does not achieve its full growth potential, resulting in low birthweight infants. IUGR represents one of the leading causes of infant mortality worldwide and has been suggested to predispose newborns to a variety of diseases. This condition does not only impact human pregnancies and is a significant problem in livestock species. Thus, there is a clear need to improve the understanding of the mechanisms regulating fetal growth and to allow the development of therapeutic strategies. The articles in this Research Topic “*Intrauterine growth restriction: screening and outcomes*” describe some of the latest studies aimed to improve the understanding of the mechanisms regulating fetal growth and development.

White and Yates provided a comprehensive review to this Research Topic where they describe the phenotype of IUGR in different animal models, with a focus on placental insufficiency, fetal pathophysiology and metabolic adaptations, and inflammatory contributions to IUGR pathologies. They then reviewed the potential of supplementation with ω-3 polyunsaturated fatty acids to improve inflammation-mediated growth and metabolic deficits in IUGR offspring.

Insulin-like growth factor-1 (IGF-1) plays an essential role in promoting fetal growth and development. Previous studies have demonstrated a positive correlation between IGF-1 abundance in umbilical cord blood at parturition and birth weight, with decreased fetal IGF-1 abundance in IUGR pregnancies. Thus, IGF-1 offers significant promise as a treatment for impaired fetal growth. However, we currently have a limited understanding of how IGF-1 regulates nutrient availability to the fetus or the molecular mechanisms that IGF-1 utilizes to regulate fetal organ growth and development. Stremming et al. contributed an intricate study to this Research Topic which utilized complementary *in vitro* and *in vivo* approaches to provide new insights into the role of IGF1 in fetal development. In the first experiment, primary skeletal myoblasts from late gestation sheep fetuses treated with 10 ng/mL ovine IGF-1 (oIGF-1) showed a significant increase (22%) in proliferation. Stremming et al. then used surgically inserted catheters to infuse oIGF-1 into Day 136 ovine fetuses and demonstrated its effects on fetal whole-body and organ growth, skeletal myoblast proliferation, and hypertrophic muscle growth *in vivo*.

IUGR is a significant problem in swine production, with recent studies suggesting that 15%–25% of newborn piglets are growth restricted. IUGR piglets commonly experience increased neonatal and preweaning mortality and morbidity, with impaired organ growth and development, accompanied by alterations in metabolism and increased disease susceptibility. The study by Amdi et al. aimed to compare hepatic transcriptional responses and innate immune system function in IUGR piglets compared to normally sized piglets on postnatal Day 3. The authors demonstrated substantial alterations in the expression of transcripts with roles in metabolism and innate immune system function in the liver. Further, investigation of blood samples from these piglets demonstrated decreased expression of IL-1β in peripheral blood mononuclear cells, fewer eosinophils, increased plasma alanine aminotransferase and blood urea nitrogen, and decreased plasma glucose concentrations in IUGR piglets compared to normal-sized piglets. The authors attempted to rescue this phenotype by providing a commercially available energy-rich oral paste and colostrum supplement. However, this supplementation appeared to have minimal effects on the piglets suggesting it may not be beneficial for rescuing the IUGR phenotype in piglets.

IUGR is associated with poor postnatal muscle growth, which can have significant consequences for both humans and species of agricultural importance. Gibbs et al. contributed an interesting article to this Research Topic where IUGR lambs received a daily intramuscular injection of clenbuterol HCl (β2 adrenergic agonist) or a saline control. IUGR lambs treated with saline or clenbuterol were lighter than non-IUGR lambs on Days 30 and 60. However, the clenbuterol injections improved average daily weight gain in the IUGR lambs. Analysis of muscle development revealed striking effects on muscle development in the IUGR lambs treated with clenbuterol HCl, suggesting that β2 adrenergic stimulation may be a potential therapeutic target to improve muscle growth in IUGR offspring.

Collectively, the articles in this Research Topic collection represent significant advancements to the field of intrauterine growth restriction and it is hoped that these findings serve as a platform for future research in this field.

